# Hemodynamic changes following injection of local anesthetics with different concentrations of epinephrine during simple tooth extraction: A prospective randomized clinical trial

**DOI:** 10.4317/jced.52321

**Published:** 2015-10-01

**Authors:** Nedal Abu-Mostafa, Fatimah Al-Showaikhat, Fatimah Al-Shubbar, Kawther Al-Zawad, Fatimah Al-Zawad

**Affiliations:** 1 BDS. MSc. Lecturer in Oral and Maxillofacial Surgery, Riyadh Colleges of Dentistry and Pharmacy, Oral and Maxillofacial Surgery and Diagnostic Science Department, Dental Hospital (Namuthajia) Riyadh, Kingdom of Saudi Arabia; 2 BDS. Dental Intern, Riyadh Colleges of Dentistry and Pharmacy, Oral and Maxillofacial Surgery and Diagnostic Science Department, Dental Hospital (Namuthajia) Riyadh, Kingdom of Saudi Arabia

## Abstract

**Background:**

Presence of epinephrine in local anesthetic cartridge increases the duration of local anesthesia (LA), decreases the risk of toxicity, and provides hemostasis. However, the unfavorable effects are increasing heart rate (HR) and raising blood pressure (BP). The aim was to evaluate hemodynamic changes in the BP, HR, and oxygen saturation (SpO2) of normal patients undergoing tooth extraction using LA with various epinephrine concentrations.

**Material and Methods:**

A prospective randomized clinical trial was conducted on 120 patients who were divided randomly into 3 parallel groups according to the LA received. Group 1: lidocaine 2% with epinephrine 1:80,000 (L80). Group 2: articaine 4% with epinephrine 1:100,000 (A100). Group 3: articaine 4% with epinephrine 1:200,000 (A200). Inclusion criteria: normal patients whose BP < 140/90. Exclusion criteria: hypertension, cardiovascular disease, hyperthyroidism, pregnancy, and allergy to LA. BP, HR, and (SpO2) were evaluated in 3 different time points: 3 minutes before LA, 3 minutes after LA, and 3 minutes after extraction.

**Results:**

Systolic blood pressure (SBP) significantly increased after injection of L80 and continued after extraction to be significant than pre-injection. SBP significantly increased after injection of A100 then decreased after extraction. In the group of A200, SBP insignificantly decreased after injection then increased after extraction. The increasing of SBP between time point 1and 2 was significantly higher in G1 than G3 (*p*=0.014). Diastolic blood pressure decreased after LA in the 3 groups; however it was significant only with L80, then increased after extraction for all.

**Conclusions:**

The changings of DBP, HR and SpO2 after anesthesia and extraction showed no significant difference among the three groups. However, A200 had significant lesser effect on SBP than L80 and the least effect on other parameters. Therefore, A200 is considered safer than L80 and A100 and is recommended for LA before teeth extraction in normal patient.

** Key words:**Local anesthesia, lidocaine, epinephrine 1:80,000, articaine, epinephrine 1:100,000, epinephrine 1:200,000, tooth extraction.

## Introduction

The use of local anesthetics in combination with vasoconstrictor agents is justified in dentistry. Doing so counteracts the local vasodilation effect of local anesthetic agents and delays its absorption into the cardiovascular system. These effects are beneficial in increasing the duration of local anesthesia and diminishing the risk of toxicity and also provide hemostasis during surgery (1).

Lidocaine is an amide local anesthetic that is used extensively for pain control since its pharmacokinetic characteristics and low toxicity make it safe for use in dental practice (2).

Also, articaine hydrochloride (HCl) is an amide local anesthetic, but it has the advantage of substitution of the aromatic ring with a thiophenic ring that increases the liposolubility of the drug as well as its potency. Moreover, articaine is the only amide LA containing an ester group in its molecular structure, thus allowing metabolization of the drug by both plasma esterases and liver (3).

Epinephrine is the main vasoconstrictor used in dentistry (1). The predominant action of epinephrine is on β receptors; however, it also effects on both α and β receptors. The vasoconstriction action of epinephrine depends on stimulating α1 receptors in perip-heral blood vessels (4). Stimulating of β 1receptors by epinephrine increases heart rate and raises blood pressure (5). On the other hand, pain during dental treatment can trigger the release of endogenous catecholamines, which, in turn, can give rise to hemodynamic changes, such as an increase in blood pressure and heart rate, and may even produce arrhythmia (6).

This study aimed to evaluate hemodynamic changes, including blood pressure, heart rate, and oxygen saturation, in normal patients following administration of local anesthetic cartridges containing three different concentrations of epinephrine: lidocaine 2% with epinephrine 1:80,000, articaine 4% with epinephrine 1:100,000, and articaine 4% with epinephrine 1:200,000.

## Material and Methods

This prospective randomized clinical trial was carried out from October 1, 2014 to January 1, 2015 in the Colleges’ Dental Hospital. One hundred-twenty patients who had single simple tooth extraction were included in the study. Dental extractions were performed by qualified dental interns under close supervision of surgery instructors. Sample size was estimated depending on a power calculation. At level of significance α = 0.05 with estimated standard deviation 1.2 and power 0.9, the sample size from each group should be at least 38.

Inclusion criteria: patients who weren’t diagnosed as hypertensive and their blood pressure <140/90 mm Hg. Exclusion criteria: hypertension, cardiovascular disease, sickle cell anemia, congenital methemoglobinemia, hyperthyroidism, pregnancy, breastfeeding, allergy to local anesthetics, any contraindications to epinephrine, and if the extraction required more than 2 local anesthetic cartridges or the duration exceeded 30 minutes.

The study followed the World Medical Association Declaration of Helsinki and was registered in the Colleges Research Center (USRP/2013/159). Study aims and procedures were explained to the included patients before signing the informed consents. Patients’ information was documented in questionnaire papers about the name, age, gender, mobile number, file number.

Patients were divided randomly into three parallel groups; each of them contained 40 patients. One hundred-twenty questionnaires contained three groups were mixed. Each group had 40 questionnaires presented a study group and its number was written (1, 2 and 3). Random distribution was achieved by asking the patient to choose one questionnaire and the type of local anesthetic was selected accordingly. Group 1: received 2 cartridges of lidociane 2% with epinephrine 1:80,000 (L 80). Group 2: received 2 cartridges of articaine 4% with epinephrine 1:100,000 (A 100). Group 3: receive 2 cartridges of articaine 4% with epinephrine 1:200,000 (A 200).

All procedures in the study were performed in morning sessions with relaxed atmosphere and no preoperative anxiolytic medications were prescribed. On the dental chair, a pulse oximeter (Merlin medical® Pulse Oximeter) was applied to the left index finger of the patient then heart rate and oxygen saturation were recorded. Blood pressure was measured by an electronic sphygmomanometer (OMRON® Automatic Blood Pressure Monitor). Three minutes later, aspiration was done followed by injection of 2 cartridges (each of 1.8 ml) of local anesthesia selected according to the group. If blood was encountered in the syringe, the clinician had to withdraw the needle, replace the cartridge, and repeat the aspiration then do the injection. Three minutes after anesthesia; blood pressure, oxygen saturation, and heart rate were measured again. The last measurements were taken three minutes after extraction for the same hemodynamic parameters. The differences of the four parameters between the 1st time point and the 2nd time point were calculated and compared among the three groups. Similar calculation was done for the difference between the 1st and 3rd time point. Data analyses were performed by statistical software SPSS version 22.0 for Windows.

## Results

One hundred-twenty patients were included in the study; 51 were males (42.5%) and 69 (57%) were females. The mean age for all patients was (36.34 years). The patients were divided into three groups, every group included 40 patients. The mean age for G1, G2 and G3 were (38.45 years), (35.17 years) and (35.40 years) respectively. The data was analyzed by one-way repeated measures ANOVA.

Infiltration injections were used in 72 cases (60%) whereas nerve blocks were used in 48 cases (40%). Positive aspiration occurred 10 times (8.3%). Eight cases occurred with nerve blocks (16.7%) and 2 with infiltration injections (2.8%). The difference was statistically significant (*p*=0.014) according to Chi-square test.

The means of hemodynamic changes in systolic blood pressure, diastolic blood pressure, heart rate, and oxygen saturation are available in  Table 1.

**Table 1:**
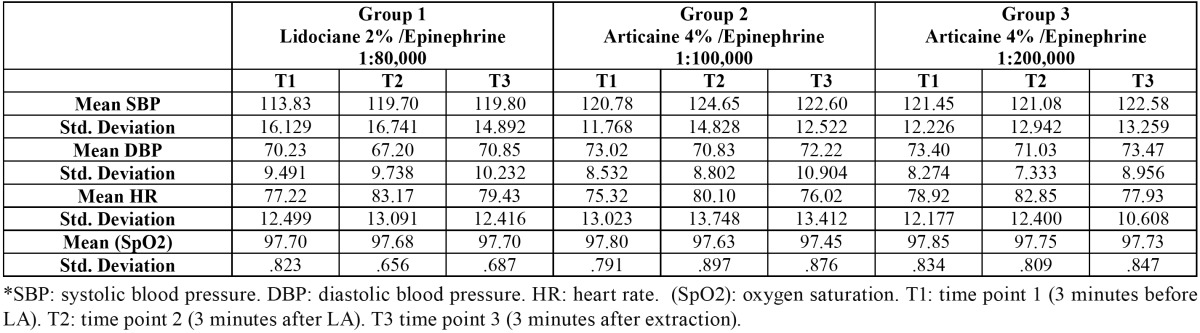
The means of systolic blood pressure, diastolic blood pressure, heart rate, and oxygen saturations for the three groups in the three time points.

**Figure 1:**
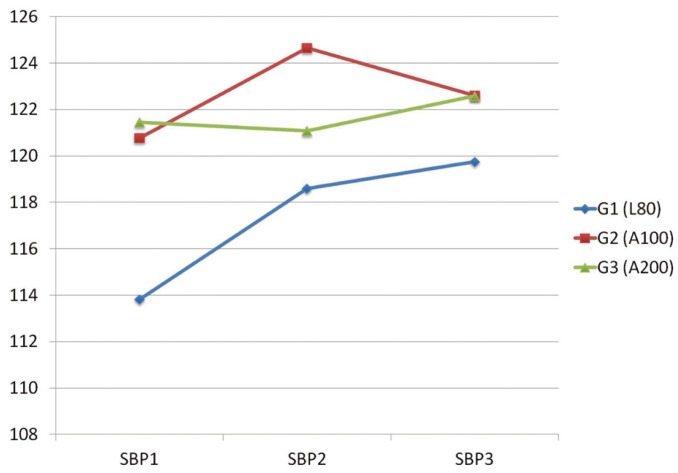
Mean Systolic Blood Pressure (SBP) measurements in the three different time points (SBP1, SBP2 and SBP3)* for the three groups of patients. *SBP1: systolic blood pressure 3 minutes before LA injection. SBP2: systolic blood pressure 3 minutes after LA injection. SBP3: systolic blood pressure 3 minutes after extraction.

The mean of systolic blood pressure significantly increased 3 minutes after injection of L80 (SBP2) (*p*=0.012) then continued within 3 minutes after extraction (SBP3) to be significant than before injection (*p*=0.006). In G2, systolic blood pressure significantly increased 3 minutes after injection of L100 (SBP2) (*p*=0.024) then decreased 3 minutes after extraction (SBP3). In G3, systolic blood pressure insignificantly decreased 3 minutes after injection (SBP2) (*p*=1.000) then increased after extraction (SBP3) (Fig. 1).

**Figure 2:**
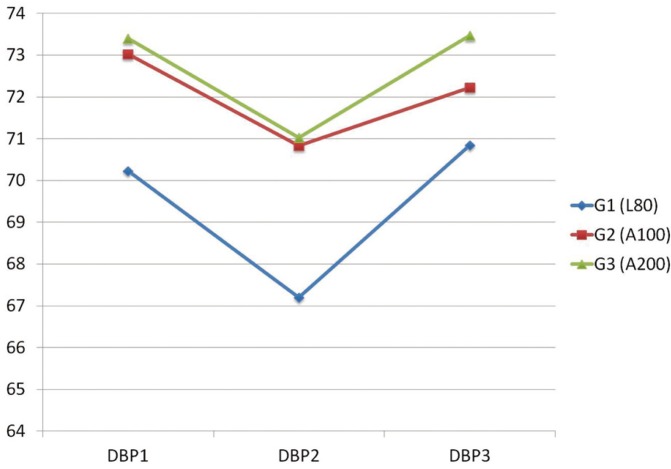
Mean Diastolic Blood Pressure (DBP) measurements in three different time points (DBP1, DBP2 and DBP3)* for the three groups of patients. *DBP1: diastolic blood pressure 3 minutes before LA injection. DBP2: diastolic blood pressure 3 minutes after LA injection. DBP3: diastolic blood pressure 3 minutes after extraction.

The mean of diastolic blood pressure decreased 3 minutes after injection (DBP2) in the 3 groups; however, it was significant only with L80 (*p*=0.010). Three minutes after extraction, diastolic blood pressure (DBP3) increased in the three groups (Fig. 2).

**Figure 3:**
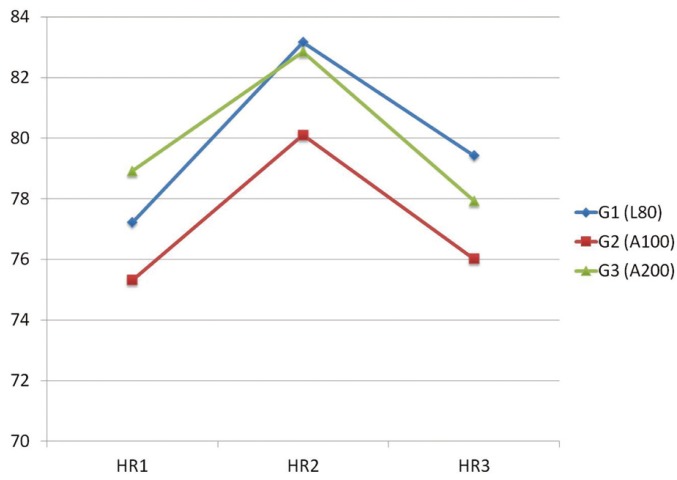
Mean Heart Rate (HR) measurements in the three different time points (HR1, HR2 and HR3)* for the three groups of patients. *HR1: heart rate 3 minutes before LA injection. HR2: heart rate 3 minutes after LA injection. HR3: heart rate 3 minutes after extraction.

The mean of heart rate significantly increased 3 minutes after local anesthetic injection (HR2) in G1, G2, and G3. *P* values respectively were (*p*=0.000), (*p*=0.000) and (*p*=0.001). Heart rate decreased 3 minutes after extraction in all groups (HR3) (Fig. 3).

The mean of oxygen saturation decreased 3 minutes after local anesthetic injection (SpO2)2 in all groups but the significance presented only in G2 (*p*=0.014). After extraction, oxygen saturation further decreased in G2 and G3 (SpO2)3 whereas it increased in G1 to take the same level of (SpO2)1.

The difference between SBP1 and SBP2 was significantly higher in G1 than G3 (*p*=0.014) according to one-way ANOVA: Post Hoc Multiple Comparisons test. Among the three groups, there were no significant differences on the other parameters between the three time points. These differences are available in  Table 2.

**Table 2:**
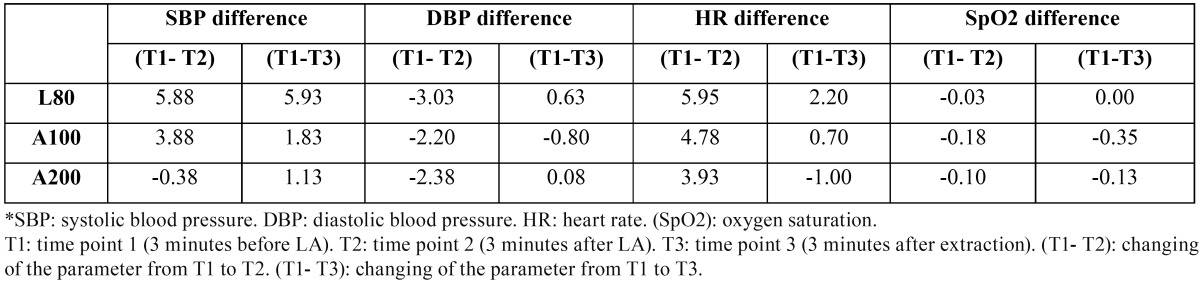
The differences of hemodynamic parameters between the three time points.

## Discussion

This study addressed the effect of three different concentrations of epinephrine on blood pressure, heart rate, and oxygen saturation of normal patients who had simple tooth extractions. The measurements of these parameters were performed at 3-minute intervals because epinephrine produces its maximum action 3 minutes after injection (6).

Aspiration before injection of local anesthesia is essential to avoid intravascular injection, which is responsible for important variations of hemodynamic changes. Positive aspiration occurs when a stream of blood rises through the cartridge with sufficient strength to mix with the anesthetic solution. The percentage of blood aspirations reported in the literature is highly variable (3.6 % – 22%) (7). In this study, aspiration was done prior to injection of the local anesthesia in all cases. Positive aspiration occurred 10 times which presented 8.3% of cases; however, it was more likely to occur with nerve block (16.7%) as compared with infiltration (2.8%) and the difference was statistically significant (*p*=0.014).

Reviewing the literature revealed that multiple studies have evaluated the effect of local anesthetics with different epinephrine concentrations on hemodynamic parameters. A clinical trial conducted by Hersh EV *et al.* in 2006 (8) compared the cardiovascular effects of A100 and A200. They used 11.9 ml (7 cartridges) which was near the maximum recommended dose of articaine (7 cartridges or 476 mg). Significant elevation of systolic blood pressure was reported at 10 minutes after injection in patients who received A100 as compared with A200.

In our results, a significant increase in systolic blood pressure presented after injection and after extraction in the group who received L80 whereas, the group of A100 had significant increase only after injection. On the other hand, A200 was associated with a decrease in systolic blood pressure after injection, which was found to be non-significant. Significant increase of systolic blood pressure after injection was reported with L80 as compared with the group of A200 (*p*=0.014). Additionally, we observed that diastolic blood pressure decreased after the local anesthetic injection in the 3 groups, but it was significant only with L80. In the same way, Hersh EV *et al.* found diastolic blood pressure tended to decrease with both formulations of local anesthetics for the first 30 minutes after injection. The decrease of diastolic blood pressure referred to the stimulation of ß2 receptors by epinephrine that leads to vasodilation of blood vessels in the skeletal muscles (1).

In contrast to our finding, in 2007, Santos CF *et al.* (9) did not find a significant change in the measurements of systolic and diastolic blood pressure with injection of A100 or A200 for third molar surgery. Harmonious results were achieved by de Morais HH *et al.* (10-12) when they performed multiple comparison studies for hemodynamic changes following injection of different local anesthetics with epinephrine: (L100 and A200), (L100 and A100), and (A100 and A200). They found no significant changes of hemodynamic parameters in normal patients following injection of these types of local anesthetics.

A study performed by Kämmerer PW *et al.* in 2014 (13) compared pulpal anesthesia after infiltration of 5 local anesthetic solutions: A100, A200, A300, A400, and Articaine plain. They pointed out that infiltration anesthesia by these solutions did not affect heart rate, blood pressure, or oxygen saturation.

The contradiction between our findings and the other studies (9-12) might be attributed to the different amounts of local anesthetics used. We used 3.6 mL of local anesthesia; L80, A100, and A200, which contained 0.045 mg, 0.036 mg, and 0.018 mg of epinephrine, respectively. The other studies used 2.7 ml of local anesthesia containing 0.027 mg or 0.0135 mg of epinephrine. Also, Kämmerer PW *et al.* (13) used only 1.7 ml of local anesthesia. Small volumes of epinephrine seem to have relatively tran-sient cardiovascular effects in healthy people (9). On the other hand, the volumes of epinephrine used in our study, or larger volumes as used by Hersh EV *et al.* (8), which contained (0.119 mg or 0.0595) mg of epinephrine, most likely caused further enhancement of alpha and beta1-adrenergic receptors that contributed to this variation in hemodynamic changes.

With regard to the heart rate, Hersh EV *et al.* (8) and de Morais HH *et al.* (12) found an increase in heart rate after administration of local anesthesia in both groups; however, A100 combined with a significant rise as compared with A200. In the same way, de Morais HH *et al.* (11) reported greater increase in heart rate associated L100 versus A200 and the difference was statistically significant. Our study recorded a significant increase in heart rate after injection of L80, A100, and A200, but the difference was not significant. Santos CF *et al.* (9) found no significant increase in heart rate and the variation was not affected by the local anesthetic used.

We found that oxygen saturation decreased after injection in the 3 groups; however, it was only significant with A100. In contrast, Santos CF *et al.* (9) and de Morais HH *et al.* (11) reported an increase in oxygen saturation after injection of A100 and A200.

In the literature, variation of epinephrine concentrations seemed to have insignificant effect on the efficacy of local anesthesia. It was found that L80 and L200 have similar success rates for inferior alveolar nerve block (14). Also, a non-significant difference was noticed between the efficacy of L80 and A100 on the pulp following infiltration injection (15). Likewise, A100 and A200 showed similar degree of anesthesia on the pulp following infiltration, nerve block, (16,17) and intra-osseous injections (18). Moreover, the two formulations produced comparable anesthesia for periodontal surgery (19) as well as anesthesia required for lower third molar extraction regarding: anesthetic properties, intraoperative hemostasis, and lack of influence on hemodynamic parameters (8,9).

Despite the fact that aspiration procedure can avoid intravascular injection, false-negative results are not uncommon (20). This may occur when the needle bevel has direct contact against the vascular endothelium that blocks the needle lumen on aspiration. Another probable cause is the collapse of a minor vessel by extremely intense aspiration (7). Accordingly, less vasoconstrictor in the solution could be safer especially for patients with cardiovascular disease (21). Consequently, it was suggested by some inves-tigators to use A200 instead of A100 for pulpal anesthesia, (16) and lower third molar extraction (8,9,12).

Variable epinephrine concentrations used in the study differently influenced hemodynamic parameters. L80, which contained the highest concentration of epinephrine, significantly affected systolic and diastolic blood pressure, and heart rate. A100 significantly affected systolic blood pressure, heart rate. The significant influence of A200 was limited on heart rate however this effect was less than others.

Based on our results, the differences of diastolic blood pressure, heart rate and oxygen saturation after anesthesia and after extraction showed no significant difference among the three groups. However, A200 had significant lesser effect on systolic blood pressure than L80 and the least effect on other parameters. Therefore, A200 is considered safer than L80 and A100 and is recommended for local anesthesia before teeth extraction in normal patient.
